# Serum *P*-Cresyl Sulfate Levels Correlate with Peripheral Arterial Disease in Hypertensive Patients

**DOI:** 10.3390/diagnostics15091097

**Published:** 2025-04-25

**Authors:** Yahn-Bor Chern, Jen-Pi Tsai, Bang-Gee Hsu, Chin-Hung Liu, Ji-Hung Wang

**Affiliations:** 1 Division of Nephrology, Department of Internal Medicine, Yuan’s General Hospital, Kaohsiung 80249, Taiwan; 2 Division of Nephrology, Department of Internal Medicine, Dalin Tzu Chi Hospital, Buddhist Tzu Chi Medical Foundation, Chiayi 62247, Taiwan; 3 School of Medicine, Tzu Chi University, Hualien 97004, Taiwan; 4 Division of Nephrology, Hualien Tzu Chi Hospital, Buddhist Tzu Chi Medical Foundation, Hualien 97004, Taiwan; 5 Graduate Institute of Clinical Pharmacy, School of Medicine, Tzu Chi University, Hualien 97004, Taiwan; 6 School of Pharmacy, Tzu Chi University, Hualien 97004, Taiwan; 7 Division of Cardiology, Hualien Tzu Chi Hospital, Buddhist Tzu Chi Medical Foundation, Hualien 97004, Taiwan

**Keywords:** C-reactive protein, *p*-cresyl sulfate, hypertension, peripheral artery disease, ankle–brachial index, diabetes mellitus

## Abstract

**Background/Objectives:** *p*-Cresyl sulfate (PCS) is implicated in inflammation, oxidative stress and vascular dysfunction. Hypertension is a major risk factor for peripheral arterial disease (PAD), which is linked to increased mortality in patients with hypertension. This study aimed to evaluate the association between serum PCS levels and PAD in hypertension cases. **Methods**: We analyzed fasting blood samples and clinical data from 105 patients with hypertension in a cardiovascular outpatient clinic. Serum PCS levels were quantified using high-performance liquid chromatography–mass spectrometry. Ankle–brachial index (ABI) was measured using an automated oscillometric device; ABI < 0.9 indicated PAD. **Results**: A total of 24 patients (22.9%) had PAD. The PAD group had a higher prevalence of diabetes mellitus (*p* = 0.026), elevated serum C-reactive protein (CRP) levels (*p* < 0.001) and increased PCS levels (*p* = 0.002) than the normal ABI group. Multivariate logistic regression showed that PCS (odds ratio [OR]: 1.154, 95% confidence interval [CI]: 1.013–1.315, *p* = 0.031) and CRP (per 0.1 mg/dL increase, OR: 1.649, 95% CI: 1.138–2.389, *p* = 0.008) were independently associated with PAD. According to Spearman’s correlation analysis, log-transformed PCS (log-PCS) levels negatively correlated with left or right ABI (*p* = 0.001 and *p* = 0.004, respectively) and estimated glomerular filtration rate (*p* = 0.001) but positively correlated with log-CRP (*p* = 0.024). **Conclusions**: Elevated serum PCS and CRP levels are significantly associated with PAD in patients with hypertension, suggesting the potential role of PCS in PAD pathogenesis.

## 1. Introduction

Peripheral arterial disease (PAD) is characterized by peripheral artery narrowing with subsequent reduction in blood flow to distal extremities, caused mainly by advanced atherosclerosis [1]. More importantly, PAD has been viewed as a risk equivalent of atherosclerotic cardiovascular disease (ASCVD) and a significant risk factor for predicting cardiovascular mortality [2]. Hypertension is a well-established major risk factor for ASCVD, as well as PAD, but its importance in these diseases cannot be stressed enough [3,4,5,6,7,8]. Nonetheless, a worldwide pooled analysis revealed the stable global age-standardized prevalence of hypertension [9]. In 2015, the prevalence of PAD was 5.6% [10]. Owing to the significant burden of PAD and its associated risk factors, an approach that can better detect PAD development in hypertension cases is needed.

*p*-Cresyl sulfate (PCS) has been extensively studied as a protein-bound uremic toxin derived from the intake of aromatic amino acids, mainly tyrosine and phenylalanine, that are subsequently processed by intestinal bacteria into phenolic end products [11]. Regarding toxicity on the human cardiovascular system, elevated PCS levels have been linked to various cardiovascular diseases [12,13], including peripheral arterial stiffness [14], coronary artery disease (CAD) [15,16,17], arrhythmia [18] and left ventricular dysfunction [19]. Increased PCS levels were reported to be associated with a low ankle–brachial index (ABI) and vascular access failure events in patients undergoing hemodialysis [20]. However, the relationship between PCS and PAD in patients with hypertension remains unclear, particularly in outpatients with a range of renal statuses. By investigating whether PCS is associated with PAD in this population regardless of the renal status, our study aimed to explore whether PCS could be another biomarker that can be integrated into current risk stratification models and enhance early detection strategies for PAD to improve cardiovascular outcomes in patients with hypertension.

## 2. Materials and Methods

### 2.1. Patients

We recruited 105 patients with hypertension from the cardiovascular outpatient department of a medical center in Hualien, Taiwan, between July 2020 and December 2020. The exclusion criteria were as follows: recent acute coronary syndrome within 3 months, acute decompensated heart failure, acute stroke within 3 months, malignancy, limb amputation, acute infection during blood sampling, ABI > 1.3, or patient refusal to provide informed consent for the study. This study obtained approval from the Research Ethics Committee, Hualien Tzu Chi Hospital, Buddhist Tzu Chi Medical Foundation (IRB108-219-A). All participants provided their written informed consent to participate in this study. After resting for at least 10 min, our trained staff measured the participants’ morning blood pressure (BP) using standard mercury sphygmomanometers with appropriate cuff sizes. Systolic BP (SBP) and diastolic BP (DBP) values were measured thrice at a 5 min interval and were averaged for the analysis. In this study, hypertension was defined as SBP ≥ 140 mm Hg and/or DBP ≥ 90 mmHg or intake of antihypertensive drugs in the past 2 weeks according to the *Eighth Joint National Committee (JNC 8)* guidelines. Moreover, diabetes mellitus (DM) was defined as fasting plasma glucose levels ≥ 126 mg/dL or intake of hypoglycemic agents.

### 2.2. Anthropometric Analysis

We measured all patients’ anthropometric variables in the morning after overnight fasting. Body weight and height measurements were recorded to the nearest 0.5 kg and 0.5 cm, respectively. Additionally, the body mass index was calculated as weight (kg) divided by the square of height (m^2^).

### 2.3. Biochemical Investigations

Immediately after collection, fasting blood samples (approximately 5 mL) were centrifuged at 3000× *g* for 10 min. The serum levels of blood urea nitrogen, creatinine, fasting glucose, total cholesterol (TCH), triglycerides (TG), high-density lipoprotein cholesterol (HDL-C), low-density lipoprotein cholesterol (LDL-C), total calcium, phosphorus and C-reactive protein (CRP) were checked using an autoanalyzer (Siemens Advia 1800; Siemens Healthcare GmbH, Henkestr, Erlangen, Germany) [21]. To measure the serum levels of human intact parathyroid hormone (iPTH), we used a commercially available enzyme-linked immunosorbent assay (Abcam, Cambridge, MA, USA) [21]. For calculating the estimated glomerular filtration rate (eGFR), we applied the chronic kidney disease epidemiology collaboration equation.

### 2.4. Determination of Serum PCS Levels by High-Performance Liquid Chromatography–Mass Spectrometry

A Waters e2695 high-performance liquid chromatography system integrated with a mass spectrometer (ACQUITY QDa, Waters Corporation, Milford, MA, USA) was used in measuring serum PCS levels in this study [14]. As for the analytical column, we used the Phenomenex Luna^®^ C18 (2) (5 µ, 250 × 4.60 mm, 100 Å) with the following operational parameters: column temperature, 40 °C; flow rate, 0.8 mL/min; an injection volume, 30 µL. In the mobile phase, a binary gradient was implemented: the initial composition (95% [A] water with 0.1% formic acid/5% [B] methanol with 0.1% formic acid) remained constant for 1 min; subsequently, solvent B was incrementally increased to 70% over a span of 12 min and held constant for an additional 2 min. For column re-equilibration, solvent B was diminished to 50% within 1 min and sustained at this level for 2 min.

The liquid chromatography–mass spectrometry (LC-MS) gradient conditions were modified, given that the pretreated samples were concurrently analyzed. The specifications of the instrument were as follows: desolvation temperature, 600 °C; capillary voltage, 0.8 kV; sample cone voltage, 15.0 V. The mass spectrometer functioned in full scan mode, covering a range of 50–450 *m*/*z* for positive ionization and 100–350 *m*/*z* for negative ionization. We employed a single-ion recording mode to monitor the specific masses of each compound (PCS: 187.0 *m*/*z*). Data acquisition and processing were conducted using the Empower^®^ 3.0 software (Waters Corporation, Milford, MA, USA). The retention time observed for PCs was approximately 16.56 min. In quantifying endogenous compounds, we measured and compared the peak areas against the calibration curve derived from the standard solutions. All the determination coefficients of linearity (*r*^2^) exceeded 0.995. The LC-MS single-ion recording mode was utilized for analyzing individual ions.

### 2.5. ABI Measurements

BP was measured using the oscillometric technique on both the upper and lower extremities, with each measurement taken thrice while the participant was in a supine position, generating data from the brachial, dorsalis pedis and posterior tibial arteries (VaSera VS-1000, Fukuda Denshi, Tokyo, Japan) [22]. ABI was computed as the ratio of the highest recorded SBP in the dorsalis pedis or posterior tibial artery at either the right or left ankle to that in the brachial artery of either upper limb. Continuous electrocardiographic monitoring was performed for over 15 min. Patients exhibiting a reduced ABI of 0.9 in either the left or right extremity were considered to have PAD, as previously mentioned [22].

### 2.6. Strategies to Avoid Bias

Several strategies were applied to reduce the risk of bias in this study. Selection bias was minimized by strict inclusion and exclusion criteria to make the study population reflect hypertensive patients in general. Recruitment was performed in a systematic manner without over-representation of certain subgroups by consecutively enrolling all eligible participants. We used validated methods and protocols to standardize data collection procedures to minimize potential information bias. Serum PCS concentrations were determined by standard laboratory methods and ABI values were assessed by trained staff members using Doppler ultrasound according to prevailing clinical protocols. Third, to minimize potential bias in how results are considered, we performed a thorough review of the literature in order to contextualize our findings with respect to related evidence while also considering the potential for publication bias.

### 2.7. Statistical Analysis

We performed a power analysis to determine whether our sample size was sufficient to detect statistically significant correlations between serum PCS levels and PAD, based on previously published findings of vascular markers and PAD [23,24]. At an α of 0.05, and 80% statistical power to detect a medium correlation coefficient (r = 0.30), which was of clinical significance in our context, the required sample size for this current study was calculated [25]. As a minimum sample size to provide sufficient power to detect any such correlation our calculations indicated at least 85 participants would be required. Our study population of 105 hypertensive patients was greater than this threshold and provided adequate statistical power. Post hoc power analysis confirmed that our sample size provided 86% power to detect the correlation observed with log-PCS and left ABI (r = −0.321, *p* = 0.001) and 78% power for the correlation with right ABI (r = −0.281, *p* = 0.004). These outcomes confirm our findings to be sufficiently reliable and suggest that the number of enrolled patients was sufficient for adequate statistical analysis.

The normality of data distribution was assessed using the Kolmogorov–Smirnov test. Continuous variables that are normally distributed are presented as means ± standard deviation, whereas those that are not normally distributed are presented as medians with interquartile ranges; comparisons between patients were conducted using the two-tailed Student’s independent *t*-test and the Mann–Whitney *U* test, respectively. Categorical variables are expressed as counts with corresponding percentages and they were analyzed using the χ^2^ test. Variables demonstrating a significant association with PAD were subsequently examined through multivariable logistic regression analysis. We also utilized the receiver operating characteristic (ROC) curve to calculate the area under the curve (AUC) and to ascertain the serum levels of PCS and CRP as predictors of PAD in individuals with hypertension. In light of the nonnormally distributed variables, we applied a base 10 logarithmic transformation to facilitate normality. The correlation between log-PCS left ABI, right ABI values and clinical variables was evaluated using Spearman’s rank correlation coefficient. All statistical data were analyzed using the SPSS software for Windows (version 19.0; SPSS, Chicago, IL, USA). A *p*-value of 0.05 was considered statistically significant.

## 3. Results

Table 1 delineates the baseline characteristics of the cohort comprising 105 individuals with hypertension, stratified into normal (*n* = 81) and low (*n* = 24) ABI categories. Within this cohort, 71 patients (67.6%) were male, and the prevalence rates of DM and CAD were 31.4% (*n* = 33) and 66.7% (*n* = 70), respectively. The prevalence of DM was significantly elevated in the low ABI group compared with that in the normal ABI group (*p* = 0.026). Additionally, the low ABI group had markedly higher levels of CRP and PCS than the normal ABI group (*p* < 0.001 and *p* = 0.002, respectively). Conversely, SBP and DBP, sex distribution, CAD presence, or antihypertensive medication use showed no statistically significant differences between the two groups.

**Figure 1:**
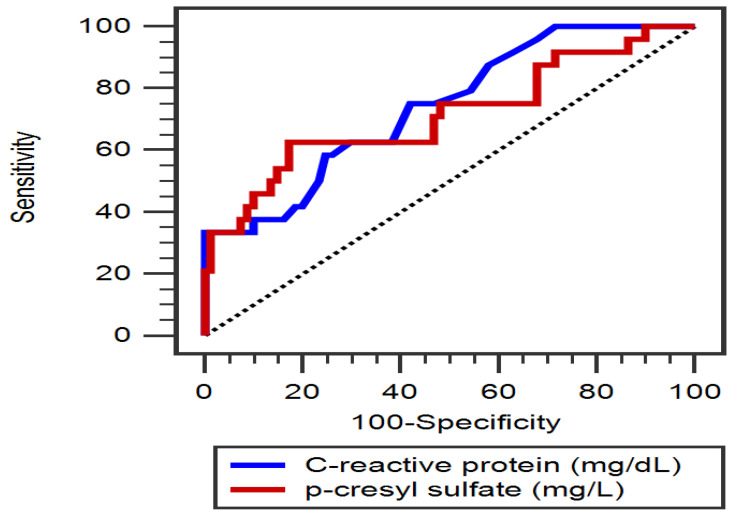
The area under the receiver operating characteristic curve indicates the diagnostic power of serum total *p*-cresyl sulfate level and C-reactive protein level for predicting peripheral artery disease among 105 hypertensive patients.

A multivariate logistic regression analysis was conducted after the adjustment for significant variables (*p* < 0.2) correlated with PAD. These variables included DM, BMI, TCH, eGFR, CRP and PCS levels. Among patients with hypertension, the serum levels of CRP (each 0.1 mg/dL increment, odds ratio [OR]: 1.649, 95% confidence interval [CI]: 1.138–2.389, *p* = 0.008) and PCS (OR: 1.154, 95% CI: 1.013–1.315, *p* = 0.031) were identified as independent predictors for PAD (Table 2). Furthermore, the ROC curve for PAD prediction indicated that the AUC values for PCS and CRP were 0.714 (95% CI, 0.618–0.798; *p* = 0.002) and 0.739 (95% CI, 0.645–0.820; *p* < 0.001), respectively (Figure 1).

Table 3 shows the association between serum log-PCS and various clinical variables through Spearman’s correlation analysis. The left and right ABI values revealed a negative correlation with log-PCS (*r* = −0.321; *p* = 0.001 and *r* = −0.281, *p* = 0.004, respectively). Furthermore, serum log-PCS positively correlated with log-CRP (*r* = 0.220, *p* = 0.024) but negatively correlated with estimated eGFR (*r* = −0.325, *p* = 0.001). Likewise, the left and right ABI values negatively correlated with log-CRP (*r* = −0.382 and *r* = −0.446, *p* < 0.001, respectively). Notably, the right ABI values positively correlated with eGFR (*r* = 0.199, *p* = 0.042).

## 4. Discussion

This study demonstrated that among the recruited patients with hypertension visiting our cardiovascular outpatient department, significantly higher prevalence rates of DM and higher levels of PCS and CRP were noted in those diagnosed with PAD measured by ABI. In the subsequent multivariable logistic regression analysis, higher serum PCS and CRP levels independently correlated with PAD presence. Furthermore, Spearman’s correlation analysis revealed that the log-PCS levels negatively correlated with the left or right ABI values and eGFR and positively correlated with the log-CRP.

Results such as higher DM prevalence and CRP levels in patients with hypertension with PAD, as well as the independent correlation of CRP levels with PAD, align well with current evidence. First, DM has been well recognized as one of the risk factors of ASCVD, including PAD [2,26,27]. As for the role of inflammation in PAD pathogenesis, CRP has been found to be a biomarker that can not only predict the presence but also the severity of PAD [26,28,29,30]. However, some authors argued that although patients with PAD exhibit elevated CRP levels, such increase may be more of a reflection of an underlying systemic inflammation resulting from various traditional and nontraditional ASCVD risk factors rather than causally linked to PAD development [31]. Therefore, some experts suggested combining CRP with other biomarkers to predict and risk-stratify PAD more accurately [32].

Considering the positive correlation between PCS levels and PAD presence, the underlying mechanism could be the involvement of PCS in atherosclerosis and vascular calcification [33,34]. Various in vitro and in vivo studies demonstrated that PCS induces reactive oxygen species (ROS) [35], activates the renin–angiotensin–aldosterone system [11], stimulates and damages endothelial cells [13,35], causes vascular smooth muscles to migrate and proliferate [33] and interacts with chemotactic proteins and complement interleukin-1 receptors leading to inflammation [36]. Thus, PCS may have the capacity to initiate and accelerate the process of atherosclerosis, which constitutes a significant risk factor for PAD. Additionally, PCS reportedly promotes osteogenesis in arterial smooth muscle cells; osteogenesis can contribute to vascular calcification and could explain the positive correlation between PCS levels and arterial stiffness observed in patients with chronic kidney disease (CKD) [14,37]. Overlapping risk factors characterize arterial stiffness and PAD and exhibit a complex interrelationship; in fact, some experts regarded arterial stiffness as a precursory lesion of PAD. Regarding the identified negative association between PCS and eGFR, research indicates that diminished eGFR results in impaired tubular secretion, which represents a principal pathway for the elimination of protein-bound uremic toxins such as PCS [11,38,39]. Moreover, metabolic acidosis, altered protein assimilation, gut microbiota changes and DM, which are commonly found in patients with CKD, could contribute to increased PCS levels [11,17,40]. Lastly, the right ABI positively correlated with eGFR. The clinical significance and potential implications of this result require further well-designed studies to specifically identify the optimal cutoff value of ABI for risk-stratifying PAD presence in patients with hypertension.

This study has several limitations that need to be addressed. First, the study is cross-sectional in design; thus, we cannot establish a causal relationship between elevated PCS levels and PAD. Second, while CRP levels are recognized as a marker of inflammation and PAD severity, their elevation may signify various underlying atherosclerotic risk factors instead of serving as a direct contributor to PAD pathogenesis. Third, the present study was limited solely to hypertensive patients who were asymptomatic for carotid artery disease and PAD. As a result, data regarding claudication and carotid disease were not obtained, and thus, symptomatic PAD or carotid artery involvement could not be evaluated. Asymptomatic PAD is a prevalent condition that frequently goes undiagnosed even though it is associated with considerable cardiovascular risk. We did not include symptomatic patients, which may limit the generalizability of our findings to other PAD presentations in the wider population. Additional studies should include both symptomatic and asymptomatic patients, as we indicate an indirect relationship between vascular markers and the presence of PAD. Another issue to be considered is that using ABI as the primary diagnostic tool for PAD may underestimate disease prevalence in hypertensive patients with medial artery calcification, a common feature in CKD and diabetes. Further studies must include adjunctive techniques like toe–brachial index or imaging modalities to better establish the diagnosis of PAD in this high-risk population. Finally, our participants were recruited from a single center; hence, they may not represent the broader hypertensive population. Considering these limitations, further studies with larger and more diverse populations with hypertension are required to clarify the potential mechanics of PCS, inflammation and PAD pathogenesis.

## 5. Conclusions

This study demonstrates that PCS may serve as a biomarker of evolving PAD in hypertensive patients. In a multivariate logistic regression analysis, elevated serum levels of PCS and CRP are independent predictors of PAD in hypertensive patients. Meanwhile, PCS positively correlates with CRP and PAD presence in this patient population and inversely correlates with eGFR. PCS showed an area under the curve (AUC) of 0.714, establishing it as a suitable diagnostic for PAD in hypertensive patients. Our findings show the possibility of PCS as a promising biomarker for early identification and risk stratification of PAD in a clinical setting, thereby potentially enabling timely intervention and subsequent appropriate follow-up management to improve patient outcomes.

## Figures and Tables

**Table 1:** The clinical variables of the 105 hypertensive patients in the normal or low ankle brachial index group.

Characteristics	All Patients (*n* = 105)	Control Group (*n* = 81)	Low ABI Group (*n* = 24)	*p* Value
Age (years)	65.07 ± 10.41	64.51 ± 9.80	66.96 ± 12.29	0.313
Height (cm)	161.01 ± 8.63	161.41 ± 8.18	159.67 ± 10.08	0.386
Body weight (kg)	69.53 ± 12.81	68.93 ± 12.23	71.58 ± 14.71	0.375
Body mass index (kg/m^2^)	26.73 ± 3.82	26.36 ± 3.60	27.96 ± 4.35	0.071
Left ankle-brachial index	1.09 (1.01–1.13)	1.11 (1.07–1.16)	0.89 (0.83–0.94)	<0.001 *
Right ankle-brachial index	1.07 (1.00–1.13)	1.09 (1.05–1.14)	0.87 (0.83–0.93)	<0.001 *
Systolic blood pressure (mmHg)	131.95 ± 16.70	132.02 ±17.13	131.71 ± 15.52	0.936
Diastolic blood pressure (mmHg)	74.50 ± 10.17	74.60 ± 9.85	74.13 ± 11.40	0.840
Total cholesterol (mg/dL)	170.94 ± 41.04	167.83 ± 40.10	181.46 ± 43.31	0.154
Triglyceride (mg/dL)	128.00 (91.00–172.50)	119.00 (88.50–179.50)	141.50 (122.50–164.25)	0.262
HDL-C (mg/dL)	44.52 ± 12.59	44.32 ± 12.51	45.21 ± 13.09	0.763
LDL-C (mg/dL)	99.88 ± 31.83	98.04 ± 32.70	106.08 ± 28.47	0.279
Fasting glucose (mg/dL)	107.00 (95.00–133.50)	106.00 (94.50–131.50)	114.00 (97.00–163.50)	0.312
Blood urea nitrogen (mg/dL)	17.02 ± 5.07	16.79 ± 4.11	17.79 ± 7.52	0.398
Creatinine (mg/dL)	1.13 ± 0.30	1.10 ± 0.27	1.23 ± 0.39	0.054
eGFR (mL/min)	66.89 ± 18.94	68.69 ± 17.75	60.80 ± 21.84	0.073
Total calcium (mg/dL)	9.14 ± 0.37	9.14 ± 0.39	9.13 ± 0.30	0.919
Phosphorus (mg/dL)	3.49 ± 0.52	3.50 ± 0.52	3.48 ± 0.51	0.860
iPTH (pg/mL)	48.10 (33.75–65.25)	47.30 (33.35–62.25)	51.50 (34.68–75.08)	0.296
CRP (mg/dL)	0.20 (0.15–0.27)	0.19 (0.14–0.25)	0.26 (0.20–0.81)	<0.001 *
Total *p*-cresyl sulfate (mg/L)	6.91 (5.02–10.55)	6.59 (4.53–8.32)	11.02 (5.70–17.93)	0.002 *
Male, *n* (%)	71 (67.6)	56 (69.1)	15 (62.5)	0.542
Diabetes mellitus, *n* (%)	33 (31.4)	21 (25.9)	12 (50.0)	0.026 *
Coronary artery disease, *n* (%)	70 (66.7)	52 (64.2)	18 (75.0)	0.324
Hyperlipidemia	86 (81.9)	68 (84)	18 (75)	0.319
Stable congestive heart failure	16 (15.2)	11 (13.6)	5 (20.8)	0.388
Stroke	5 (4.8)	3 (3.7)	2 (8.3)	0.352
ACE inhibitor use, *n* (%)	34 (32.5)	28 (34.6)	6 (25.0)	0.379
ARB use, *n* (%)	49 (46.7)	37 (45.7)	12 (50.0)	0.709
β-blocker use, *n* (%)	57 (54.3)	42 (51.90)	15 (62.5)	0.358
CCB use, *n* (%)	40 (38.1)	33 (40.7)	7 (29.2)	0.305
Statin use, *n* (%)	60 (57.1)	46 (56.8)	14 (58.3)	0.893
Fibrate use, *n* (%)	21 (20.0)	14 (17.3)	7 (29.2)	0.201
Aspirin, *n* (%)	63 (60.0)	48 (59.3)	15 (62.5)	0.776
Clopidogrel, *n* (%)	30 (28.6)	25 (30.9)	5 (20.8)	0.339

Values for continuous variables are shown as mean ± standard deviation after analysis by Student’s *t*-test; variables not normally distributed are shown as median and interquartile range after analysis by the Mann–Whitney *U* test; values are presented as number (%) and analysis after analysis by the chi-square test. ABI, ankle-brachial index; HDL-C, high-density lipoprotein cholesterol; LDL-C, low-density lipoprotein cholesterol; eGFR, estimated glomerular filtration rate; iPTH, intact parathyroid hormone; CRP, C-reactive protein; ACE, angiotensin-converting enzyme; ARB, angiotensin-receptor blocker; CCB, calcium-channel blocker. * *p* < 0.05 was considered statistically significant.

**Table 2:** Multivariable logistic regression analysis of the factors correlated to peripheral artery disease among the 105 hypertensive patients.

Variables	Odds Ratio	95% Confidence Interval	*p* Value
Total *p*-cresyl sulfate, 1 mg/L	1.154	1.013–1.315	0.031 *
C-reactive protein, 0.1 mg/dL	1.649	1.138–2.389	0.008 *
Diabetes mellitus, present	2.095	0.609–7.199	0.240
Body mass index, 1 kg/m^2^	1.098	0.936–1.288	0.249
eGFR, 1 mL/min	0.995	0.960–1.030	0.766
Total cholesterol, 1 mg/dL	1.004	0.991–1.017	0.547

eGFR, estimated glomerular filtration rate. Data was analyzed using the multivariate logistic regression analysis (adopted factors: diabetes mellitus, body mass index, estimated glomerular filtration rate, total cholesterol, C-reactive protein and total *p*-cresyl sulfate). * *p* < 0.05 was considered statistically significant.

**Table 3:** Spearman correlation coefficients between left ABI, right ABI, log-endocan and clinical variables in 164 patients with chronic kidney disease.

Variables	Left Log-ABI		Right Log-ABI		Log-PCS (mg/L)	
	Spearman Coefficient of Correlation	*p* Value	Spearman Coefficient of Correlation	*p* Value	Spearman Coefficient of Correlation	*p* Value
Age (years)	–0.112	0.255	–0.187	0.056	0.031	0.752
Body mass index (kg/m^2^)	–0.139	0.157	–0.057	0.561	0.049	0.621
Left ABI	**—**	**—**	0.690	<0.001 *	–0.321	0.001 *
Right ABI	0.690	<0.001 *	**—**	**—**	–0.281	0.004 *
Log-PCS (mg/L)	–0.321	0.001 *	–0.281	0.004 *	**—**	**—**
SBP (mmHg)	0.042	0.669	–0.036	0.718	0.011	0.910
DBP (mmHg)	0.060	0.545	0.040	0.685	–0.009	0.926
Total cholesterol (mg/dL)	–0.179	0.068	–0.056	0.574	0.104	0.292
Log-Triglyceride (mg/dL)	–0.035	0.723	0.024	0.811	0.093	0.347
HDL-C (mg/dL)	–0.092	0.349	–0.120	0.224	–0.079	0.421
LDL-C (mg/dL)	–0.125	0.203	0.006	0.948	0.100	0.308
Log-Glucose (mg/dL)	0.004	0.972	–0.042	0.673	–0.005	0.961
eGFR (mL/min)	0.149	0.129	0.199	0.042 *	–0.325	0.001 *
Total calcium (mg/dL)	–0.005	0.964	–0.041	0.675	0.139	0.159
Phosphorus (mg/dL)	0.001	0.992	–0.022	0.824	0.156	0.111
Log-iPTH (pg/mL)	–0.153	0.119	–0.051	0.605	–0.061	0.576
Log-CRP (mg/L)	–0.382	<0.001 *	–0.446	<0.001 *	0.220	0.024 *
Age (years)	–0.112	0.255	–0.187	0.056	0.031	0.752

Data of PCS, triglyceride, glucose, iPTH and CRP levels showed skewed distribution and, therefore, were log-transformed before analysis. Analysis of data was performed using the Spearman correlation analysis. ABI, ankle-brachial index; PCS, *p*-cresyl sulfate; SBP, systolic blood pressure; DBP, diastolic blood pressure; HDL-C, high-density lipoprotein cholesterol; LDL-C, low-density lipoprotein cholesterol; eGFR, estimated glomerular filtration rate; iPTH, intact parathyroid hormone; CRP, C-reactive protein. * *p* < 0.05 was considered statistically significant (two-tailed).

## Data Availability

Upon request, the corresponding author can provide the data utilized in this study.
